# Roots Withstanding their Environment: Exploiting Root System Architecture Responses to Abiotic Stress to Improve Crop Tolerance

**DOI:** 10.3389/fpls.2016.01335

**Published:** 2016-08-31

**Authors:** Iko T. Koevoets, Jan Henk Venema, J. Theo. M. Elzenga, Christa Testerink

**Affiliations:** ^1^Swammerdam Institute for Life Sciences, Plant Cell Biology, University of AmsterdamAmsterdam, Netherlands; ^2^Genomics Research in Ecology and Evolution in Nature – Plant Physiology, Groningen Institute for Evolutionary Life Sciences, University of GroningenGroningen, Netherlands

**Keywords:** abiotic stress tolerance, root system architecture (RSA), salinity, drought, nutrient limitation, flooding, temperature stress tolerance, crop breeding

## Abstract

To face future challenges in crop production dictated by global climate changes, breeders and plant researchers collaborate to develop productive crops that are able to withstand a wide range of biotic and abiotic stresses. However, crop selection is often focused on shoot performance alone, as observation of root properties is more complex and asks for artificial and extensive phenotyping platforms. In addition, most root research focuses on development, while a direct link to the functionality of plasticity in root development for tolerance is often lacking. In this paper we review the currently known root system architecture (RSA) responses in *Arabidopsis* and a number of crop species to a range of abiotic stresses, including nutrient limitation, drought, salinity, flooding, and extreme temperatures. For each of these stresses, the key molecular and cellular mechanisms underlying the RSA response are highlighted. To explore the relevance for crop selection, we especially review and discuss studies linking root architectural responses to stress tolerance. This will provide a first step toward understanding the relevance of adaptive root development for a plant’s response to its environment. We suggest that functional evidence on the role of root plasticity will support breeders in their efforts to include root properties in their current selection pipeline for abiotic stress tolerance, aimed to improve the robustness of crops.

## Introduction

### From Optimal to Suboptimal Conditions – Closing the Yield Gap

The world population is growing rapidly and this is accompanied by an increased food demand. In past decades, this growing food demand has been addressed by plant breeding consistent with optimal conditions for plant growth. In agricultural practices, the use of fertilizers, irrigation, pesticides, and other inputs can create these optimal conditions on the short-term. However, increasing evidence exists for the negative consequences of these practices on the long-term.

First of all, irrigation accounts for almost 70% of all freshwater usage in the world (FAO and ITPS, 2015). Freshwater scarcity is a big threat to the human population and the current water usage for agriculture is not sustainable (Rosegrant et al., 2009). Furthermore, irrigation causes salinization of soils (Smedema and Shiati, 2002) and increases leaching of fertilizer. This leaching, together with excess use of fertilizer and deep tilling leads to higher greenhouse gas emissions (Snyder et al., 2009).

These problems illustrate the unsustainability of creating the optimal conditions our crops are selected for. In addition, climate change will further increase this challenge. Agriculture will have to deal with growing crops under suboptimal conditions, creating a gap between the yield potential and the currently reached yield – the so-called yield gap. An extensive research field tries to map the current yield gap (Lobell et al., 2009; Licker et al., 2010; Van Ittersum et al., 2013) with much focus on improving land management practices (Lobell et al., 2009; Mueller et al., 2012). In concert, plant breeding is shifting from creating “specialist” cultivars that require optimal conditions for their performance toward creating “robust” cultivars that can perform optimal in a broad range of suboptimal conditions, with the ultimate goal of closing the yield gap.

Crop yield is driven by the combination of climate, soil, management, and genetics. Under optimal circumstances the soil provides plants with stability, water, and nutrients. However, soils are heterogeneous environments, strongly influenced by outside factors. Nutrient deficiency, drought, salinity, flooding, and temperature are major drivers of the current and future yield gap. Researchers and breeders work together to develop crops that are able to withstand these stresses (as reviewed in Mickelbart et al., 2015). However, current crop selection is mainly focused on the shoot, whereas most major drivers of the yield gap affect soil properties, directly influencing the root system. This paper will therefore focus on the potential of optimizing root systems for improving crop abiotic stress tolerance.

### Roots Bridging the Yield Gap

Breeding efforts to improve crop yield are in general focused on aboveground, shoot-related phenotypes, whereas the roots as ‘hidden half’ of the plant are still an under-utilized source of crop improvement (Den Herder et al., 2010; Wachsman et al., 2015). Trials aimed to select for new cultivars with improved crop yield are in general performed under optimal nutrient concentrations, which has often led to selection for smaller and less plastic roots (White et al., 2013). Moreover, modern cultivars develop in general faster and the earlier initiation of shoot sinks stimulates the investment of biomass into the shoots rather than into the roots. Modern wheat cultivars indeed have smaller root sizes and root:shoot ratios than older ones (Siddique et al., 1990; Waines and Ehdaie, 2007). Given the crucial role roots play in the establishment and performance of plants, researchers have started ‘the second green revolution’ to explore the possibility of yield improvements through optimization of root systems (Lynch, 2007).

**FIGURE 1:**
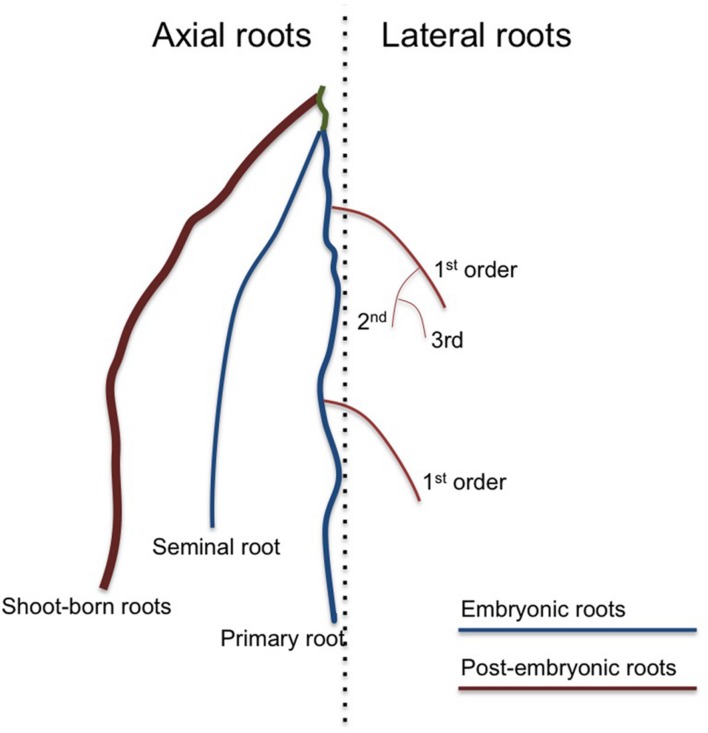
**An overview of the different root types that together form the root system**. A dicot root system consists only of one primary root and several orders of lateral roots. In addition, dicots can produce special stress-induced shoot-born roots called adventitious roots. A monocot root system produces additional axial roots, which can be separated in embryonic seminal roots and non-embryonic shoot-born roots. There are several types of shoot-borne roots, such as nodal and crown roots, often distinguished by the exact place they develop and their increasing thickness. In monocots, the primary and seminal roots are especially important during early seedling establishment, but shoot-born roots soon take over and are responsible for most of the water and nutrient uptake. All axial root types can produce several orders of lateral roots.

**FIGURE 2:**
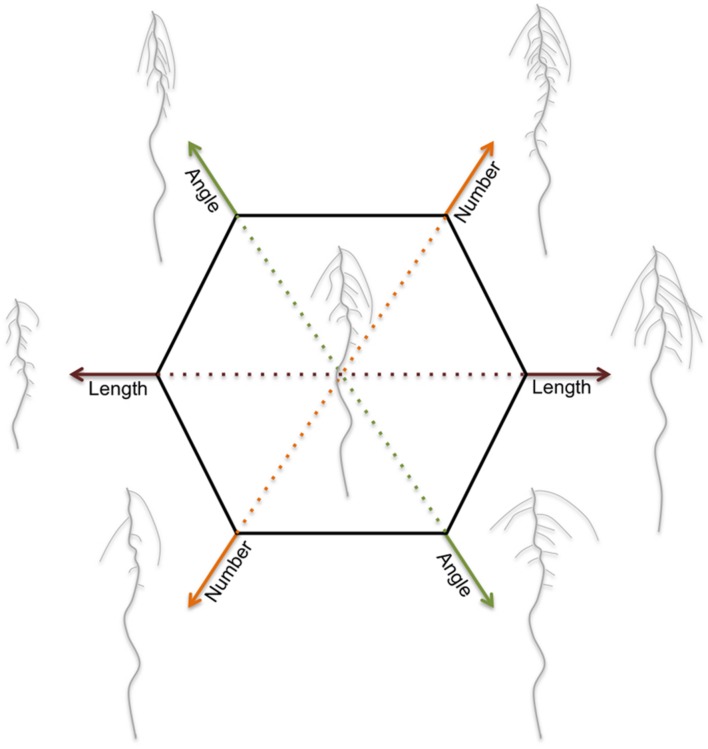
**RSA is defined as the spatial configuration of root components and determines the soil volume that can be explored by the roots**. Dicot roots consist of a main root and several orders of lateral roots. Monocot roots contain in addition seminal roots and shoot-borne roots. Each plant species has genetically defined limits to its RSA. Within these limits, the RSA is plastic and external (abiotic stress) factors modulate the length, number, positioning and angle of root components. The RSA plasticity varies strongly among and within plant species. This figure illustrates the modulations in RSA for a typical dicot root system.

Because water and nutrients are not evenly distributed in the soil, the spatial arrangement of the root system is crucial for optimal use of the available resources. This spatial arrangement of the root and its components is referred to as root system architecture (RSA). Length, number, positioning, and angle of root components (as described in **Figure 1**) together determine RSA (**Figure 2**). These traits determine the soil volume that is explored. In addition, the root surface area depends on root hair development and root diameter. The ability to adjust RSA is an important aspect of plant performance and its plasticity to a large variety of abiotic conditions (Smith and De Smet, 2012). Root development is guided by environmental information that is integrated into decisions regarding how fast and in which direction to grow, and where and when to develop new lateral roots (Malamy, 2005). The limits of root system plasticity are determined by intrinsic pathways governed by genetic components (Pigliucci, 2005; Smith and De Smet, 2012; Gifford et al., 2013; Jung and McCouch, 2013). Understanding the development and architecture of roots, as well its plasticity, holds thus great potential for stabilizing the productivity under suboptimal conditions in the root environment (de Dorlodot et al., 2007; Den Herder et al., 2010; Zhu et al., 2011). Although, plants are capable of adjusting a wide range of developmental and molecular processes in the root to cope with abiotic stress, this review will mainly focus on the plasticity of RSA, their proposed adaptive values, and its use in the selection and breeding of more robust crops.

## Nutrient Limitation: Adapting RSA for Optimal Foraging

Plants use macronutrients as the basis of proteins and nucleic acids. Especially the availability of phosphorus (P) and nitrogen (N) determine plant performance. Other nutrients are used as co-factors for enzymes or to drive membrane transport. Complications in nutrient acquisition can arise because of nutrient shortage in the soil, but other factors such as pH, the balance of different nutrients and soil composition also play a role. For examples, high salinity can decrease the solubility and thus availability of phosphate (Grattan and Grieve, 1998; Hu and Schmidhalter, 2005).

Nutrient deficiencies are responsible for the major part of currently observed yield gaps worldwide. Mueller et al. (2012) estimated that for 73% of the areas with a yield gap bigger than 25%, solely improving nutrient balances in the soil could close this gap. This illustrates the impact of nutrient imbalances and deficiencies on plant productivity. If we also consider the high use of fertilizer in agriculture, improving plants’ capability of dealing with nutrient deficiencies and increasing their of nutrient acquisition is of major importance.

Nutrients are distributed heterogeneously and often have a strong vertical distribution pattern. Leaching on the one hand and plant cycling on the other hand influence the nutrient distribution pattern. Leaching is caused by vertical water flow and takes nutrients down to lower soil layers, were water flow decreases and nutrients accumulate. Plant cycling is based on nutrients taken up from and cycled back to the soil, which causes nutrients to deplete in the root zone and accumulate in the topsoil. Horizontal distribution of nutrients is mainly dependent on the plant distribution aboveground, leading to higher nutrient accumulation underneath canopies. Vertical distribution depends on the balance between leaching and plant cycling, which differs strongly between nutrients. Low mobile nutrients with a prominent role in plant growth, such as phosphate and potassium, undergo high plant cycling, leading to topsoil accumulation. In contrast, mobile nutrients, such as nitrate and chloride, are subject to leaching leading to accumulation in deeper soils (Jobbágy and Jackson, 2001, 2004). The challenge for plants is to cope with this heterogeneous and sometimes contrasting distribution of nutrients and other resources. In agriculture, plant cycling is often reduced, due to harvesting of plant material, increasing leaching and loss of nutrients. To cope with this heterogeneity, plants can adapt their RSA to specifically forage those parts of the soils where nutrient availability is high.

Recently, RSA changes upon a wide range of nutrient deficiencies have been mapped in *Arabidopsis* growing on agar plates (Gruber et al., 2013). Each deficiency led to a distinct response in RSA development, which is consistent with the fact that not all nutrients have the same accumulation pattern and thus ask for a different response. For example, the readily available forms of the two most limiting nutrients, nitrate (NO_3_^-^) and phosphate (PO_4_^3-^), have an almost opposite accumulation pattern in the soil (Jobbágy and Jackson, 2001). Whereas immobile phosphate accumulates in the topsoil, mobile nitrate quickly leaches to deeper soils. This challenges the plant to respond differently to a deficiency of these nutrients. Fortunately, the RSA responses to these deficiencies have been mapped extensively in both *Arabidopsis* and crop species, offering us many insights in functional RSA development.

### Topsoil Foraging for Phosphate

**FIGURE 3:**
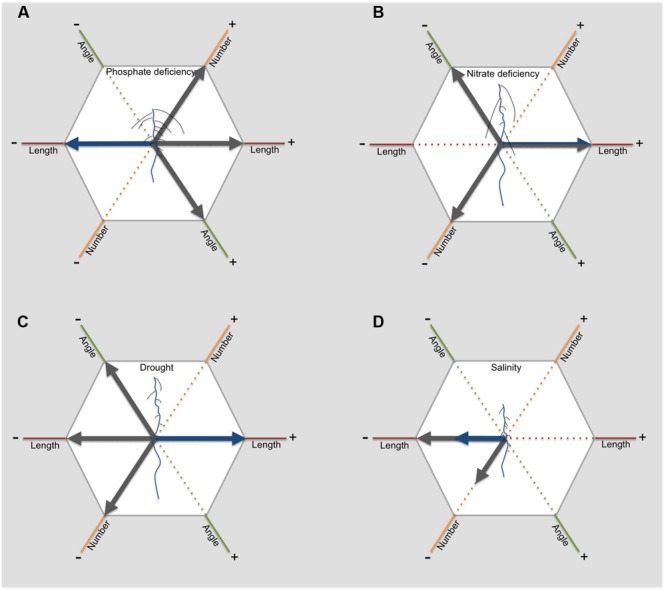
**The RSA responds to abiotic stress in different ways**. This figure illustrates for dicots how length, angle and number of primary (blue) and lateral roots (grey) change in response to phosphate deficiency **(A)**, nitrate deficiency **(B)**, drought **(C)** and salinity **(D)**. The arrows indicate an either positive (to the right) or negative effect (to the left).

Phosphate is a building block of, for example, nucleic acids and membrane phospholipids. Because of the high phosphate demand of plants, limitation in phosphate has a strong effect on plant growth (as reviewed in Péret et al., 2011; López-Arredondo et al., 2014). Efficient uptake of phosphate is therefore essential. High plant cycling, in combination with low mobility, leads to accumulation of phosphate in the topsoil. To optimally forage the soil for phosphate, plants need to develop a shallow root system (as reviewed in Lynch and Brown, 2001). The RSA response to phosphate deficiency in *Arabidopsis* is well-characterized (as reviewed by Péret et al., 2011). A strong shift from main root growth to lateral root growth is observed, which leads to a short root with a high number of long laterals (**Figure 3A**; Williamson, 2001; Linkohr et al., 2002; López-Bucio et al., 2002; Gruber et al., 2013). In addition, a strong proliferation of root hairs is observed. These changes result in a shallow root system, optimal for topsoil foraging.

For maize, a series of papers was published in which the value of certain root traits for phosphate acquisition was evaluated using a set of RILS distinctly different in these root traits. Shallow rooting maize varieties showed increased net phosphate acquisition, corrected for possible higher phosphate investments (Zhu et al., 2005b). A big screen of 242 accessions of maize on high and low phosphate availability confirmed the importance of root plasticity under low phosphate conditions (Bayuelo-Jiménez et al., 2011). Yield and biomass was increased for accessions with a higher number of nodal and lateral roots. In addition, dense root hair formation also correlated with higher biomass under low P conditions.

Shallow root system development is a result of strong investment in lateral root growth. Zhu and Lynch (2004) confirmed that in maize enhanced lateral root formation is beneficial for net phosphate acquisition. In comparison to the primary root and other components of the root system, lateral roots are cheap in terms of phosphate use. Similar results were found for enhanced seminal root growth, which is especially important for phosphate acquisition during early seedling development (Zhu et al., 2006). Several studies show that strigolactones are key regulators of both root and shoot responses to the level of available phosphate (Koltai, 2011; Ruyter-Spira et al., 2011; Mayzlish-Gati et al., 2012; Matthys et al., 2016). The effect of strigolactones on RSA depends on phosphate availability. Whereas strigolactones inhibit lateral root emergence and elongation and promote primary root elongation when phosphate is sufficient (Kapulnik et al., 2011a; Matthys et al., 2016), the opposite is observed when phosphate is depleted (Ruyter-Spira et al., 2011). Interestingly, a similar phosphate dependent effect of ABA on lateral root development has recently been observed (Kawa et al., 2016). The contrasting effect of strigolactones is a result of modulation of auxin distribution and sensitivity (Koltai et al., 2010; Ruyter-Spira et al., 2011; Mayzlish-Gati et al., 2012), both underlying the strong shift from primary to lateral root growth (López-Bucio et al., 2002; Nacry, 2005; Pérez-Torres et al., 2008a,b; Miura et al., 2011). Addition of the synthetic auxin NAA doubled expression levels of genes involved in the cell cycle specifically during phosphate starvation (Pérez-Torres et al., 2008b). Increased auxin sensitivity during phosphate starvation appears to be explained by increased expression of the auxin receptor TRANSPORT INHIBITOR RESPONSE1 (TIR1), leading to increased degradation of AUX/IAA and released repression on auxin response modules (Pérez-Torres et al., 2008a). Interestingly, strigolactones have been shown to be responsible for the increase of TIR1 expression during phosphate limitation (Mayzlish-Gati et al., 2012).

The inhibition of primary root growth in *Arabidopsis* (Col-0) in response to phosphate starvation has been shown to be strong and irreversible (Sánchez-Calderón et al., 2005). During phosphate starvation, primary root development changes drastically, shifting from indeterminate growth to determinate growth (Sánchez-Calderón et al., 2005; Kawa et al., 2016). Preceding this drastic shift, changes in the quiescence center are observed, suggesting an important role during phosphate starvation. Consistently, Svistoonoff et al. (2007) show that specifically exposing the root cap to low phosphate is sufficient to induce growth arrest in the primary root. Mutants lacking determinate growth in low phosphate conditions show reduced activation of the phosphate starvation rescue system (Sánchez-Calderón et al., 2006). These findings suggest an important role for the root cap in sensing environmental conditions.

During phosphate limitation, *Arabidopsis* develops a high number of long root hairs (Bates and Lynch, 1996). Compared to mutants lacking root hairs, wild type plants have a higher phosphate uptake resulting in more plant growth (Bates and Lynch, 2000). Gahoonia and Nielsen (1998) measured phosphate uptake of root hairs by providing the radioisotope ^32^P to root hairs of rye plants in the soil. The root hairs contributed to a substantial amount of 63% of total phosphate uptake. Consistently, a mutant of barley lacking root hairs took up half the amount of phosphate compared to the wild type (Gahoonia et al., 2001). Under low phosphate conditions, cultivars of barley with long root hairs are able to sustain high yields, whereas cultivars with short root hairs produce substantially less yield (Gahoonia and Nielsen, 2004). Interestingly, no disadvantage of root hair development under high phosphate availability is found for either *Arabidopsis* and Barley (Bates and Lynch, 2000, 2001; Gahoonia and Nielsen, 2004). As for other root traits, strigolactones seem to play a major role in the regulation of the number and length of root hairs (Koltai et al., 2010; Kapulnik et al., 2011a,b; Mayzlish-Gati et al., 2012).

Next to length and number of root components, the angle of the roots also determines whether a root system develops shallow or deep. Roots are able to sense gravity, allowing the main root to grow down into the soil, a response called gravitropism. Although lateral roots are also gravitropic, they typically show a gravitropic setpoint angle (GSA; Rosquete et al., 2013; Roychoudhry et al., 2013), resulting in non-vertical emergence from the main root (see also salinity and drought sections). Under low phosphate conditions, gravitropism could be expected to counteract development of a shallow root system ideal for topsoil foraging. In accordance, in common bean, development of a shallow root system depends on the ability to adjust the gravitropic offset angle. This ability indeed correlated with its ability to cope with low phosphate conditions (Bonser et al., 1995). Subsequent investigation of RILs with contrasting root gravitropic offset angles showed a strong correlation with phosphate acquisition and plant growth (Liao et al., 2004).

### Deep Rooting and Selective Root Placement for Nitrate

In contrast to phosphate, nitrate is highly mobile in soils and is therefore prone to leaching. In environments where nitrate is limiting, deeper soil layers can often offer nitrogen supplies. Consistently, availability of phosphate and nitrate has contrasting effects on RSA. Low nitrate availability in general limits plant growth. However, low nitrate availability does not limit primary and lateral root elongation, enabling the root system to reach deeper layers of the soil (**Figure 3B**; Linkohr et al., 2002; Gruber et al., 2013). This shift in investment results in an increase in root:shoot ratio. For maize, a monocot species, reaching greater rooting depth requires the development of a lower number of crown roots. Maize genotypes with lower crown root number showed 45% greater rooting depth, which was accompanied with higher N acquisition (Saengwilai et al., 2014). The biggest difference in N acquisition was found in deeper layers, emphasizing the importance of a deep root system for nitrogen acquisition.

Lateral root density is not affected by homogeneous nitrate limitation. Interestingly, in a heterogeneous environment, a strong increase in lateral root density in nitrate patches is observed in both *Arabidopsis* and maize (Linkohr et al., 2002; Dina in ’t Zandt et al., 2015). When plants are exposed to nitrate patches, lateral root elongation rates outside the patches were strongly decreased, indicating a shift of investment of resources. Plants are thus able to selectively place their roots to efficiently forage the soil. The mechanism of utilization of heterogeneously distributed nutrients by selective placement of lateral roots in or near nutrient enriched patches is best studied for nitrogen. However, selective root placement for a wide range of nutrients was already illustrated in 1975. A limited part of the root system of barley was exposed to high concentrations of phosphate, nitrate, ammonium, and potassium (Drew, 1975). For all of these nutrients a strong proliferation of lateral roots in the zone of high availability was observed. Growth of lateral roots in other zones was strongly limited. This emphasizes the importance of investigating this response for other nutrients.

The nitrate transporter NRT1.1 plays an important role in perceiving nitrate levels in the soil. The *nrt1.1* mutant displays no increase in lateral root proliferation in nitrate rich patches (Remans et al., 2006a), while the RSA response to homogeneous nitrate limitation is not affected in this mutant, indicating that this is not an effect of reduced nitrate uptake. Interestingly, NRT1.1 has the ability to transport auxin and this transport is inhibited by nitrate (Krouk et al., 2010). Mounier et al. (2014) showed that in nitrate patches, nitrate inhibits auxin transport by NRT1.1 out of lateral root tips and primordia, leading to auxin accumulation and stimulation of lateral root growth. Outside these patches, nitrate levels are low and NRT1.1 prevents accumulation and thus lateral root growth.

NRT1.1 has been shown to affect expression of several downstream genes involved in nitrate starvation responses, including NRT2.1 (Muños et al., 2004). NRT2.1 is a major component of high-affinity nitrate uptake in the root (Wirth et al., 2007). NRT1.1 and NRT2.1 seem to be responsible for repression of lateral root growth outside nitrate patches, based on their mutant phenotypes (Little et al., 2005; Remans et al., 2006b; Krouk et al., 2010). Nitrate starvation can trigger ethylene production, a phytohormone that influences root growth (Tian et al., 2009). NRT2.1, also induced by nitrate starvation, seems to stimulate ethylene production (Zheng et al., 2013). Conversely, ethylene inhibits NRT1.1 and NRT2.1 expression, possibly providing a negative feedback loop important for fine tuning responses (Leblanc et al., 2008; Tian et al., 2009).

## Drought: Searching for Water Supplies

Besides nutrient limitation, water limitation is the biggest driver of the yield gap. Mueller et al. (2012) have shown that in 16% of the areas with a current yield gap bigger then 25%, improving irrigation can solely close the gap. In addition, for all investigated areas improving irrigation would decrease the gap. This illustrates the importance of water availability for plants. Plants need water for transport, structure and photosynthesis among other processes. Most crops have high water requirements and are poorly drought resistant. However, irrigation is already responsible for 70% of the total use of available freshwater (FAO and ITPS, 2015). The present focus of plant breeders therefore is on improving water use efficiency of crops.

When water availability is limited, the soil osmotic potential decreases and plants are confronted with osmotic stress. Plants cannot take up water and sometimes even loose water to the soil. The high surrounding osmotic potential leads to loss of turgor, starting in the root. The combination of rapid sensing and signaling, followed by adjustments on both cellular and organ level, can enable the plant to limit water loss and survive drought stress (as reviewed in Robbins and Dinneny, 2015). Drought stress leads to distinct changes in RSA, both on whole-root system and sub-organ level.

### Whole-Root Level: Deeper Rooting for Water

Water is generally stored in deeper soil layers, because the topsoil dries more quickly. Plants that develop deeper root systems will have access to water stored in these deeper layers. Among other traits, deeper rooting has been shown to be beneficial for plant production and survival under water limiting conditions (as reviewed in Comas et al., 2013). For example, the generally deeper rooting mutant *extremely drought tolerant1 (edt1)* in *Arabidopsis* shows high drought tolerance (Yu et al., 2008). This is explained by the ectopic overexpression of the HD-ZIP transcription factor *HDG11*, which directly promotes the transcription of genes encoding cell wall loosening proteins. These proteins promote cell elongation in the root, leading to an extended root system (Xu et al., 2014). Interestingly, expression of *HDG11* in other species such as rice, poplar and cotton, also confers drought tolerance (Yu et al., 2013, 2016).

Reaching deeper soils requires a shift from investment in lateral roots to investment in axile roots (**Figure 3C**). *Arabidopsis* shows a strong inhibition of lateral root emergence and elongation when grown on agar medium containing an osmoticum, such as sorbitol or mannitol, mimicking osmotic stress (Deak and Malamy, 2005; Xiong et al., 2006). Importantly, Xiong et al. (2006) showed a possible link between inhibition of lateral root growth on agar and drought tolerance in soil. Mutants performing well under drought conditions in soil, showed high sensitivity to ABA leading to strong inhibition of lateral root length on agar media. In comparison, less tolerant mutants showed no inhibition of lateral root length. ABSCISIC ACID INSENSITIVE4 (ABI4), enhanced by ABA during drought stress, can inhibit PIN1 expression, leading to decreased polar auxin transport and decreased lateral root formation (Shkolnik-Inbar and Bar-Zvi, 2010; Rowe et al., 2016). This mode of action of ABA provides a possible mechanistic explanation for the effect of ABA on lateral root formation.

Polar auxin transport by influx and eﬄux carriers determines auxin distribution in the root, which is not only important for LR formation, but also for bending of plant organs by differentially affecting cell elongation. This bending is essential for gravitropism of the main root. Positive gravitropism, growing in the direction of gravity, orientates the root downward and enables penetration of the soil. However, other root components, such as lateral, seminal and crown roots can display very different growth angles, partly suppressing gravitropism. The angle of these roots strongly determines whether RSA develops shallow or deep. In lateral roots PINs determine auxin distribution and thus the GSA (Rosquete et al., 2013). The magnitude of the difference in auxin concentration between the upper and lower side of the lateral root determines how strong a lateral root will bend (Roychoudhry et al., 2013). As previously described, auxin transport is inhibited during drought stress due to the inhibition of PIN1 expression (Liu et al., 2015), which might facilitate increased downward bending of the roots.

In several crop species increased downward bending of the roots is correlated with drought tolerance. In rice, a strong correlation between the angle of roots and drought tolerance is observed (Kato et al., 2006). High expression of the DEEPER ROOTING1 (DRO1) gene in rice, responsible for increased downward bending of the roots by altering the auxin distribution, results in maintained high yield under drought stress (Uga et al., 2013). This example indicates that adapting RSA, in this case both using genetic and transgenic approaches, can result in increased drought tolerance. Similar to rice, the angle of seminal roots in wheat cultivars also correlates with drought tolerance (Manschadi et al., 2008). Drought tolerant wheat cultivars develop seminal roots with a narrow angle, growing deeper into the soil.

### Sub-organ Level: Hydrotropism and Hydropatterning

Although a strong vertical distribution pattern of water exists, soil heterogeneity in water content exists and sensing of available water is crucial for optimal water uptake. It has been shown that plants are able to partially repress gravitropism and grow toward water, the so-called hydrotropism response (as reviewed in Eapen et al., 2005; Takahashi et al., 2009; Cassab et al., 2013). To investigate hydrotropism in *Arabidopsis*, different growth systems have been used, in which either salt solutions or agar with sorbitol created a gradient in osmotic potential and thus a gradient in water availability. *Arabidopsis* was able to redirect growth of its main root away from a low osmotic potential and thus low water availability (Takahashi et al., 2002; Kaneyasu et al., 2007; Moriwaki et al., 2013). This moisture-driven hydrotropic response has also been observed in other species including maize (Takahashi and Scott, 1991), cucumber (Mizuno et al., 2002), and pea (Takahashi and Suge, 1991; Takahashi et al., 1996).

As described previously, the distribution of auxin, driven by polar auxin transport, has a central role in regulating bending of plant organs and response to gravity. Interestingly, hydrotropism seems to be independent from polar auxin transport, as the repression of influx and eﬄux carriers of auxin do not inhibit the response (Kaneyasu et al., 2007). Recently, auxin distribution during hydrotropism was measured with the DII-VENUS SENSOR (Shkolnik et al., 2016). Indeed, during the first 2 h of hydrotropic response, no change in auxin distribution was observed. In the presence of NPA, an inhibitor of auxin transport, hydrotropic bending was not inhibited. The involvement of auxin through changes in auxin sensitivity or biosynthesis remains ellusive due to contrasting results showing either positive, negative or no effects of inhibition of auxin responses or sensitivity (Takahashi et al., 2002; Kaneyasu et al., 2007; Shkolnik et al., 2016).

It has been shown that *Arabidopsis* roots can distinguish a wet from a dry surface and selectively favor development of roots in these wet places over development in dry places (Bao et al., 2014). These wet surfaces determine where new lateral root founder cells are formed. Deak and Malamy (2005) have shown that under dry conditions lateral root primordia develop at similar rates as under control conditions. These primordia can subsequently be rapidly induced in zones with high water availability. The combination of formation and emergence of primordia leads to specific root proliferation at sites of high water availability, so-called hydropatterning. This process seems to be independent of the major drought stress hormone, ABA (Bao et al., 2014). Further research on this new topic is required to provide more knowledge on how plant roots sense moisture and adjust RSA accordingly.

## Salinity

Salinity is a major and increasing problem for agriculture (Rengasamy, 2006). Most crop species are salt sensitive and grow poorly on salinized soils (Sairam and Tyagi, 2004; Munns et al., 2006; Munns and Tester, 2008). In 1992, the extent of salinity-affected soils was estimated at 410 billion ha. Although an adequate mapping of the current extent of salinized soils is lacking, over 100 countries are confronted with soil salinization. On a yearly basis between 0.3 and 1.5 million ha of arable land are lost to salinization and another 20–40 million ha are strongly affected by salinity (FAO and ITPS, 2015). Although some of these are naturally occurring saline soils, current observed salinization is often the result of irrigation practices. Irrigation in arid zones, accounting for approximately 40% of irrigation worldwide, mobilizes salts stored in the deeper soil layers (Smedema and Shiati, 2002). In addition, due to freshwater scarcity, an increased use of brackish irrigation increases salt levels even further. The increasing losses of arable land due to salinization ask for the development of salt tolerant crops.

Similar to drought, salinity can cause problems due to the high osmotic potential in the soil, leading to osmotic stress. In addition, salinity affects plant growth due to the toxicity of high sodium Na^+^ levels. Na^+^ toxicity especially causes problems in the shoot by inhibiting photosynthesis among other processes (Munns, 2002). Na^+^ is chemically similar to K^+^ and can interfere with processes in which K^+^ plays an essential role (Benito et al., 2014). The capacity to maintain a low Na^+^/K^+^ balance in the shoot has been shown to be closely linked to salt tolerance (Møller et al., 2009). Preventing Na^+^ transport to the shoot is thus very important. The root system is responsible for water uptake, accompanied by dissolved ions including Na^+^, and thus plays an essential role in preventing Na^+^ from entering the vascular system and reaching the shoot.

### Remodeling of the Root System during Salt Stress

Salt has a distinct effect on root growth (as reviewed in Galvan-Ampudia and Testerink, 2011). Although, low salt concentrations up to 50 mM can promote plant growth in *Arabidopsis* (Zolla et al., 2010; Zhao et al., 2011; Julkowska et al., 2014), higher salt concentrations have severe negative effects. Both primary and lateral root growth is inhibited during salt stress (**Figure 3D**; Julkowska et al., 2014). In addition, lateral root number specifically decreases in the root zone developed after exposure to salt stress (**Figure 3D**; Julkowska et al., 2014). Most studies show no effect of salt stress on lateral root density, indicating that the decrease in number of lateral roots is related to the inhibition of primary root growth (Julkowska et al., 2014).

Within seconds after exposure to salt stress, plant signaling is activated. This early signaling leads to adjustments in plant growth (as reviewed in Julkowska and Testerink, 2015), starting with a quiescence of growth in all plant organs. The quiescence phase is caused by a temporary inhibition of mitotic activity, leading to lower cell division rates (West et al., 2004). After the quiescence phase, growth recovers again. However, growth rates only recover to a certain extent, because the inhibition of the cell cycle during the quiescence phase results in fewer cells in the meristem (West et al., 2004). In addition, mature cell length is smaller in salt stressed roots.

Quiescence is induced by abscisic acid (ABA), which is rapidly up-regulated under salt stress due to the decrease in osmotic potential (Jia et al., 2002; Duan et al., 2013; Geng et al., 2013). ABA in general inhibits both gibberellin (GA) and brassinosteroid (BR) signaling (Achard et al., 2006; Gallego-Bartolome et al., 2012) and stress-induced reduction of growth has been shown to benefit the plant (Achard et al., 2006). It is thus proposed that the quiescence phase is essential to induce changes to cope with salt stress. The quiescence phase is followed by a partial growth recovery, that is mainly guided by an increase in GA and BR levels (Geng et al., 2013).

The length of the quiescence phase differs strongly between root components. Whereas quiescence in the main root takes approximately 8 h, this phase can take up to 2 days in lateral roots (Duan et al., 2013; Geng et al., 2013). In a similar way, the recovery extent of different organs differs. Although overall an inhibition of root growth is observed, there is a distinct difference between the effects of salt on primary in comparison to lateral root growth. Julkowska et al. (2014) have shown that in Col-0 the relative growth rate of the primary root was more strongly affected than the growth rate of the lateral roots. This indicates that the RSA is remodeled during salt stress. The adaptive value of this remodeling with respect to salinity tolerance is still unclear and requires further research.

In a screen of 32 *Arabidopsis* accessions, a first indication for a relation between remodeling of RSA during salt stress and salt tolerance was found (Julkowska et al., 2014). The screen revealed four distinct growth strategies during salt stress, depending on the relative inhibition of the number of lateral roots, main root and lateral root growth rates. One of these strategies was correlated with a much lower Na^+^/K^+^ level in the shoot, indicating less Na^+^ uptake and thus a higher tolerance. This strategy is characterized by a strong inhibition of lateral root growth rates, while main root growth rates and number of lateral roots are much less affected (**Figure 3D**).

Besides remodeling of the root system during salt stress, plants also show reduced gravitropism under saline conditions (Sun et al., 2007). Galvan-Ampudia et al. (2013) showed that plants can specifically redirect growth away from higher salt concentrations, a response called halotropism. This response was observed in *Arabidopsis*, tomato and sorghum seedlings, both on agar media and in soil. Similar to gravitropism, auxin redistribution is central in regulating halotropism. Endocytosis of PIN2, an auxin eﬄux carrier, at the side of high salt concentrations, redistributes auxin in the root (Galvan-Ampudia et al., 2013). The redistribution of auxin is supported by auxin-induced expression of AUX1, an auxin influx carrier (van den Berg et al., 2016). Both mathematical modeling and experimental data have shown that these processes, together with a transient PIN1 increase, are responsible for the root bending away from salt (Galvan-Ampudia et al., 2013; van den Berg et al., 2016).

Part of the salinity response is also triggered by osmotic stress and shows overlap with drought responses. However, the changes in RSA show distinct differences. For example, main root growth is strongly promoted during drought, whereas it is inhibited during salt stress. It is not well-known whether the above described quiescence phase is also displayed during drought stress. Because the osmotic component of salinity is believed to underlie this response, it is worth investigating. For halotropism and hydrotropism, although similar responses, the underlying mechanisms seem to differ. In contrast to halotropism, hydrotropism has shown to be independent of auxin transport (Kaneyasu et al., 2007). Halotropism is dependent on auxin distribution and occurs only in response to Na^+^ ions, indicating it is a specific response to high salinity (Galvan-Ampudia et al., 2013; Pierik and Testerink, 2014). For drought stress, the function of changes in RSA has been studied extensively, whereas salinity research has been more focused on the underlying mechanistic principles. In future research, studying the overlaps and differences between these stresses can benefit knowledge in both areas.

Most crop species are highly sensitive to salinity. Tomato serves as a model crop that is widely used to study how salt tolerance can be enhanced in crop species. For a wide range of vegetables, including tomato, grafting is a very effective way to increase crop resistance to biotic and abiotic stresses, without affecting above ground characteristics (see also challenge 3 in section on crop selection). For several salt sensitive commercial tomato cultivars, grafting onto rootstocks of more tolerant cultivars has positive effects on productivity when exposed to high salinity (Estañ et al., 2005; Martinez-Rodriguez et al., 2008). The Na^+^/K^+^ levels in the shoot (scions) indicated that the tolerant rootstocks prevented Na^+^ reaching the shoot, illustrating the importance of the root system for salt tolerance. Unfortunately, only little is known about RSA development of crops during salt stress. In rice, rye, and maize inhibition of root length has been observed under high salinity (Rodriguez et al., 1997; Rahman et al., 2001; Ogawa et al., 2006). Similar to *Arabidopsis*, maize shows a quiescence phase in response to exposure to high salinity, followed by recovery (Rodriguez et al., 1997). In rye, the reduction in root growth is related to a reduction in cell division and an increase in cell death (Ogawa et al., 2006). Further research on remodeling of the root system of crop species will be necessary to use our current knowledge in *Arabidopsis* to improve crop tolerance to salinity.

## Flooding: Anaerobic Stress

Already 10% of cultivated land surface is so poorly drained that waterlogging, leading to anoxic conditions in the root zone, causes crop yield losses. Twenty percent of agricultural land in Eastern Europe and the Russian Federation and 16% in the USA are too wet for optimal plant functioning (Setter and Waters, 2003). As climate change is expected to lead to more frequent heavy precipitation during the plant growth season in some areas, these problems will increase. Flooding and hypoxia impose an immediate and dramatic limitation for root functioning. Limiting the oxygen supply to root cells causes an almost instantaneous arrest of root growth (as reviewed in Gibbs et al., 1998). Switching from aerobic respiration to the glycolytic generation of ATP leads to a severe reduction in energy available for maintenance, growth and ion uptake. Of these three different functions, growth takes 20–45% of ATP generated through respiration (Veen, 1981; van der Werf et al., 1988; Poorter et al., 1991; Scheurwater et al., 1998, 1999). Balancing the demand for energy with the reduced production through glycolysis could therefore also cause limiting root growth. Arrest of root growth could, however, also be caused by accumulation of products of anaerobic metabolism. A lethal drop in pH of the cytoplasm can occur when protons accumulate in the cytoplasm and the vacuole (Gerendás and Ratcliffe, 2002). In *Phragmites australis* addition of low molecular weight monocarboxylic acids, such as acetic acid, propionic acid, butyric acid and caproic acid, and sulfide, at concentration levels that have been measured *in situ*, arrested root elongation (Armstrong and Armstrong, 2001). As the rate of root elongation is one of the most important parameters determining nutrient uptake rate (Silberbush and Barber, 1983; Dunbabin, 2006), flooding-induced inhibition of root growth ultimately would lead to nutrient limitation and negatively impact the survival of the whole plant.

One of the best-studied adaptations of plants to flooding conditions is the formation of aerenchymatic tissue in the root, which provides an alternative pathway for the supply of oxygen to the root tissue (Jackson and Armstrong, 1999; Gibberd et al., 2001; Rubinigg et al., 2002). This requires that new, well-adapted, adventitious roots are being formed (Visser et al., 1996). In these roots, axial oxygen loss can be kept to a minimum so that the root tip becomes a well-oxygenated micro-climate (Jackson and Armstrong, 1999). Most of the disadvantages for root metabolism imposed by the flooding-induced hypoxic conditions are thereby ameliorated. In monocot plants the formation of new nodal roots, replacing the old seminal roots and often containing aerenchyma, can be stimulated, leading to superficial rooting patterns (Rich and Watt, 2013). If plants are not capable of increasing their oxygen supply through aerenchymous conducts in the root or by placing new roots close to the soil surface where the oxygen level might be higher, survival of flooding is unlikely.

## Temperature

Temperature is a key abiotic factor involved in seed germination and subsequent root system development during early seedling establishment. The temperature of the soil fluctuates by sinusoidal oscillations on a diurnal scale. However, depending on soil depth, changes in soil temperature are delayed and much lower in amplitude than variations in the atmospheric temperature (Walter et al., 2009). The root-zone temperature (RZT) thus fluctuates daily, seasonally, and with soil depth (Füllner et al., 2012). Depending on the season and the time of the day, the temperature of the root environment can be significantly different than the atmospheric temperature experienced by the shoots. The RZT directly affects root development, uptake and upward transport of water and nutrients (Aroca et al., 2001), phytohormone production (Ali et al., 1996; Veselova et al., 2005), which in turn affect water status (Bloom et al., 2004), stomatal conductance (Dodd et al., 2000), photosynthesis (Hurewitz and Janes, 1983), biomass partitioning (Delucia et al., 1992; Engels, 1994), leaf (Poiré et al., 2010), and shoot growth (Venema et al., 2008; Sakamoto and Suzuki, 2015). Plant species clearly differ in their optimal temperature range for root development; e.g., oat 4–7°C (Nielsen et al., 1960), wheat 14–18°C (Porter and Gawith, 1999), pea 15–20°C (Gladish and Rost, 1993), tomato 22–25°C (Gosselin and Trudel, 1984), sunflower 25–30°C (Seiler, 1998), and cotton 32–35°C (Mcmichael et al., 1993). Root:shoot ratios usually increase under unfavorable RZTs as long as temperature limits for root development are not reached (Engels, 1994; Venema et al., 2008; Füllner et al., 2012). This adaptation in root:shoot ratio may overcome restrictions in water and nutrient uptake due to increased water viscosity and/or decreased root hydraulic conductance (Equiza et al., 2001; Aroca et al., 2012). Global climate change is likely to exacerbate plant abiotic stress in coming decades by increasing fluctuations in soil temperature and (related) water availability (Lynch and Brown, 2012). Breeding crops with a broader root-zone temperature optimum is therefore of significant importance to improve future plant performance. Improved knowledge of the key regulators for RSA optimization would support these breeding efforts.

### Temperature Effects on RSA

The exposure of both mono- and dicot plant roots to temperatures below or above their optimum temperature generally decreases (i) primary root length, (ii) lateral root density (numbers of lateral roots per unit primary root length) and (iii) the angle under which lateral roots emerge from the primary root, whereas the average lateral root length is unaffected (Mcmichael et al., 1993; Seiler, 1998; Nagel et al., 2009). In addition, roots suffering from supraoptimal temperature stress start to initiate second and third order laterals (Pardales et al., 1999) and are characterized by an increased average root diameter (Qin et al., 2007). In general, the modulating effect of sub- and supraoptimal RZTs on RSA development reduces the volume that roots may access for the uptake of water and nutrients. However, root temperature was kept spatially uniform in all these studies. Remarkably, monocot barley plants exposed to a vertical RZT gradient of 20–10°C showed increased shoot and root dry masses of 144 and 297%, respectively, and a 161% increase in root:shoot ratio compared with plants grown at a uniform RZT of 20°C (Füllner et al., 2012). Barley exposed to the vertical RZT revealed also accelerated tiller formation. The higher root biomass of plants grown at the vertical RZT gradient was not the result of longer roots but was associated with a higher proportion of thicker roots. Additionally, root systems developed under a vertical RZT gradient were much stronger concentrated in the upper 10 cm of the soil substrate gradient and their N and C concentrations were significantly lower than under uniform RZT conditions. These data clearly demonstrate that knowledge gained from experiments with uniform RZTs cannot simply be extrapolated to the field where roots experience vertical temperature gradients.

**FIGURE 4:**
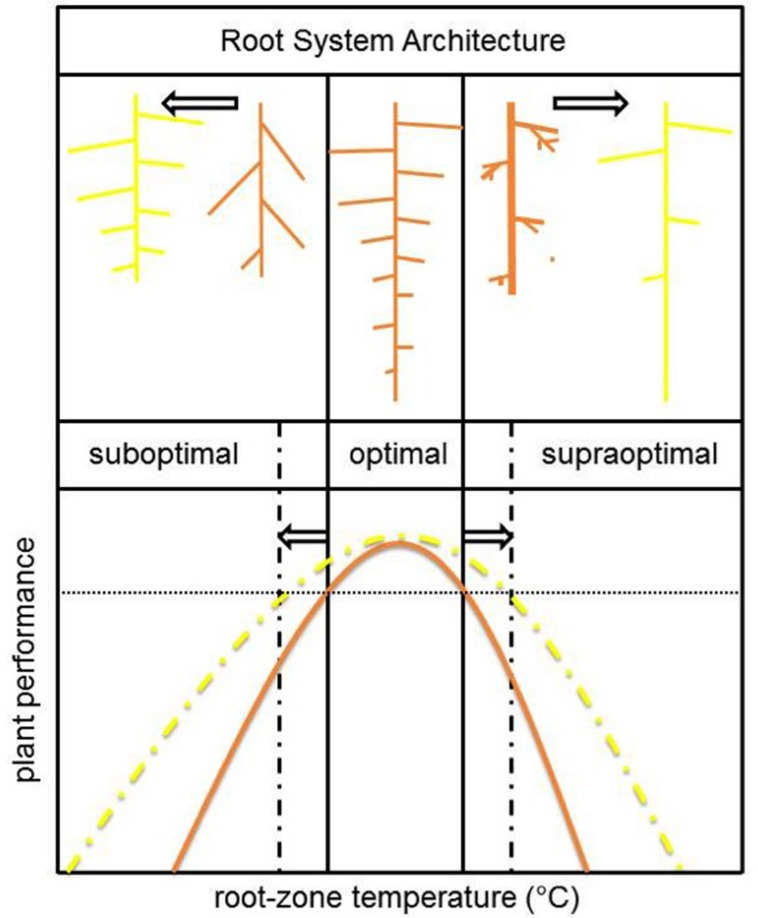
**Schematic overview of the effect of root-zone temperature on plant performance and underlying general changes in RSA (brown)**. To broaden the temperature range for optimal plant performance (yellow), plants should invest in lateral root formation (suboptimal temperature range) and/or axile root length (supraoptimal temperature range). The adaptive value of these RSA changes are, respectively, an increased root surface area to improve resource uptake capacity and drought adaptation by penetration to lower soil layers.

The temperature dependence of RSA development shows strong inter- (Mcmichael et al., 1993; Lee et al., 2009) and intraspecific variation (Seiler, 1998; Hund et al., 2007, 2008). The temperature plasticity of the RSA is most extensively studied in maize. In this monocot species, the total lateral root length correlated significantly with improved photosynthesis-related traits and dry matter accumulation at suboptimal growth temperature (Hund et al., 2007). A high density of long lateral roots was therefore regarded as a promising trait to improve early seedling vigor at suboptimal soil temperatures (Hund et al., 2008). Nevertheless, breeding has to focus on optimizing RSA over a broad range of RZTs as roots also experience temperatures in the optimal- or even supraoptimal range during the entire growth season. At high (root-zone) temperatures the development of long axile roots is of greater importance than lateral roots to facilitate appropriate water uptake from the lower soil layers in times of drought stress (Hund et al., 2008). A schematic overview of general observed effects of non-optimal temperatures on RSA and its adaptations to broaden the RZT range for optimal plant performance are presented in **Figure 4**.

To optimize RSA over a broader temperature range, Hund et al. (2012) provided the prove-of-concept that hybrids of southern dent and northern flint maize inbred lines, which contrast in temperature dependence of axile and lateral root elongation rates, showed improved rooting potential across the sum of all temperatures. Application of this heterosis effect can lead to hybrids that can perform well in a broader range of temperature conditions, thereby improving the robustness of whole-plant performance.

### Temperature Modulation of Root Elongation

The primary stunting effect of sub- and supraoptimal temperatures on RSA is caused by inhibition of root elongation (Pahlavanian and Silk, 1988; Pritchard et al., 1990; Pardales et al., 1992; Gladish and Rost, 1993; Nagel et al., 2009). In *Arabidopsis* accessions, the relative decrease in root elongation rates after transfer from 21 to 10°C were not significantly correlated with the average temperature during the growing season of the specific ecotype, suggesting that primary root growth at 10°C is not a key factor in adaptation to colder habitats (Lee et al., 2009). Within tomato, however, the relative inhibition of root elongation and root growth rates at low temperatures were indicative for the difference in chilling tolerance between domestic cultivars and high-altitude accessions of the wild tomato *Solanum habrochaites* (Zamir and Gadish, 1987; Venema et al., 2008). Dynamic changes in temperature severely affect the elongation rate of root cells rather than the length of the elongation zone (Nagel et al., 2009). In the short-term (hours), inhibition in root cell elongation by low temperature is related to a decrease in the *in vivo* extensibility of the cell wall (Pritchard et al., 1990). Gravitropism experiments with *Arabidopsis* roots demonstrated that acute cold stress (4°C) selectively inhibits the basipetal auxin transport due to blocking the intracellular trafficking of a subset of proteins that include auxin eﬄux carriers (*PIN2* and *PIN3*). As a consequence, auxin accumulates to a level at which root cell elongation is inhibited (Shibasaki et al., 2009). When plant roots have enough time to acclimate to a constant low RZT (weeks), cell elongation rates increase again and the length of the elongation zone expands (Pahlavanian and Silk, 1988). This may explain the strong linear relationship between temperature and elongation rates of both primary and later roots directly after germination and its disappearance later on during seedling establishment (Aguirrezabal and Tardieu, 1996).

Variation in root elongation rates among *Arabidopsis* accessions correlated at optimal temperature with the production rate of cells within the root meristem (Beemster et al., 2002). Cell production, in turn, was determined by variation in cell cycle duration and, to a lesser extent, by differences in the number of dividing cells. Cell production rates strongly correlated with the activity of the cyclin-dependent kinase (CDKA). Low temperature decreased the division potential of the root meristem in *Arabidopsis* by reducing both the meristem size and cell number (Zhu et al., 2015). The repression of the division potential of root meristematic cells at a suboptimal temperature of 16°C could be ascribed to a reduced accumulation of auxin in the root apex. Long-term (7 days) exposure to 16°C inhibited the expression of *PIN1*/*3*/*7* and auxin biosynthesis-related genes suggesting that auxin transport and biosynthesis both contribute to the low-temperature mediated reduction of auxin accumulation in roots tips. Root length and meristem cell number of ARABIDOPSIS RESPONSE REGULATOR 1 (*arr1-3*) and 12 (*arr12-1*) cytokinin signaling mutants were much less susceptible to low temperature than wild-type roots. This difference was related to higher *PIN1/3* expression in the mutants, which in turn resulted in a less pronounced reduction in auxin accumulation. These data, together with the results obtained with the cytokinin signaling mutant *ahp1-1 ahp2-1 ahp3*, strongly suggest the involvement of cytokinin signaling in the modulation of RSA development at low temperature (Zhu et al., 2015).

High RZT (40°C) reduced the elongation and cell production rate of Sorghum seminal roots with 14 and 26%, respectively, for every 2 days of exposure (Pardales et al., 1992). In contrast to low temperatures, the underlying inhibitory effects of high temperatures and heat stress on root elongation are poorly studied. The limited information that is available in the literature excludes the involvement of altered IAA transport or levels (Gladish et al., 2000), but supports the involvement of increased ethylene levels (Qin et al., 2007). Inhibitors of ethylene biosynthesis partly alleviated the effect of high RZT on root elongation, stomatal conductance and shoot water status, however, they failed in ameliorating the negative effects on photosynthesis and biomass accumulation. This points to a non-stomatal limitation of photosynthesis mediated by high temperature-induced changes in nutrient uptake (Qin et al., 2007).

## Crop Selection on RSA: The Challenges

This review presents a number of examples in which plasticity of RSA traits considerably impact a plant’s capability to cope with one or more abiotic stresses. These examples emphasize the great potential that selection on RSA traits holds for crop improvement. However, the aboveground focus of crop selection is not without reason. In this concluding section we will discuss three major challenges breeders face when applying selection for RSA in their crop improvement programs and possible ways to tackle these.

### Challenge 1: High Throughput Belowground Screening

The most prominent challenge for crop selection on RSA is uncovering the hidden world of plant roots. Whether crops are grown in fields or in greenhouses, roots are usually grown in a substrate, which prevents easily screening their properties. The growth substrate also greatly affects how roots develop. The currently most common method to investigate RSA, growth on agar medium, is very artificial. Most often roots grow in light, with an excess of sucrose, in 2D and the humidity inside the Petri dish is almost saturated. Effort is taken to improve this system, for example by shielding the roots from light (Silva-Navas et al., 2015). Although agar media provide an easy, adequate and cheap method that can be used for research on *Arabidopsis* in the lab, its use in crop selection is not straightforward.

In the last decade a wide range of new and improved methods to research roots in a more natural environment have been developed (as reviewed in Zhu et al., 2011; Downie et al., 2015; Judd et al., 2015; Kuijken et al., 2015). Most systems are based on either a transparent growth medium or a medium from which the roots can easily be removed without damage. Agar and other gel-like mediums are suited for imaging during growth, although the resistance of the medium influences root growth and the humidity in these substances is very high. A good alternative is hydroponics, in which the root is growing inside a nutrient solution (Tocquin et al., 2003; Chen et al., 2011; Le Marié et al., 2014; Mathieu et al., 2015). Hydroponics is also used in greenhouse culture, making it highly relevant for crop selection. This system also eases harvesting roots for different purposes and measuring exudates of roots. However, roots develop very differently, because resistance is lacking and humidity and nutrients are dispersed homogeneously. In addition, a good supply of oxygen is essential to prevent oxidative stress. A third alternative which is also very promising for automated imaging is aeroponics (Zobel et al., 1976; Ritter et al., 2001). In this system roots are grown in water-saturated air created by for example spraying with water and nutrients. This system lacks any material to grow in, which eases imaging. However, without much resistance, roots grow very vast and can have problems extending their root system to the sides against gravity. Last, root systems can be grown inside soil, which is of course most realistic for field crops and many greenhouse crops. However, non-destructive imaging inside soil asks for imaging methods that reach further than a simple camera. Several groups have recently reported the use of X-ray and MRI scans to image roots inside the soil (Mooney et al., 2012; Mairhofer et al., 2013; Metzner et al., 2015; Wang et al., 2015). Although these methods are more expensive, they offer great opportunities for automated imaging. An alternative method is GLO-Roots, based on luminescence genes expressed inside roots (Rellán-Álvarez et al., 2015). This system visualizes the root system through a thin layer of soil. For fundamental research labs, this is more feasible and also offers the opportunities to image the expression of certain genes in the root system. For root breeding, this is less interesting, because the plants are genetically modified and grown in 2D systems.

As more methods come available to study the root system and also methods are developed suitable for high-throughput screening of root systems, the need for good root image analysis software is growing. A wide range of root image analysis software exists (as reviewed in Lobet et al., 2013; Spalding and Miller, 2013; Kuijken et al., 2015). These tools range from automated to non-automated. For a limited amount of data, non-automated software prevents mistakes and gives the user a lot of freedom. However, the analysis is very time consuming and is therefore not suited for large datasets. Automated software can analyze a large dataset rapidly, but especially in complex root systems the analysis is limited to global data such as rooting depth and width. In semi-automated software, such as SmartRoot (Lobet et al., 2011) and EZ-rhizo (Armengaud et al., 2009), the level of user interactions is greater to ensure a lesser degree of analysis errors. Again, this will be more time consuming for larger root systems. In addition, when observing very large root systems, it is even hard to separate roots by eye. Therefore, the development of improved methods of root image analysis has high priority for the field.

Above described methods are all suited for 2D images of root systems. When simplifying root growth to a 2D system, spatial orientation of roots gets lost. Therefore, new methods such as growing roots in gel cylinders (Iyer-Pascuzzi et al., 2010) and using X-ray to image through soil will offer sophisticated opportunities to grow and image roots in 3D (as reviewed in Piñeros et al., 2016). Although only limited options for reconstructing and analyzing 3D images are currently available, it might eventually be easier to analyze 3D than 2D images, because overlapping and clumping together of roots will be much less common. Developing a good automated imaging analysis set-up of root systems can offer great advancements in crop selection and would be an entirely feasible investment for breeding companies.

### Challenge 2: Dealing with the Complexity of Interacting Stresses

Although the complexity of the combination of different biotic and abiotic stresses is not restricted to the root system, it does make selecting on RSA more challenging. The described RSA responses are mostly known for single stresses and some of the responses are very contrasting. A good example for this is that in drought-tolerant cultivars with a deep-rooting water-conserving phenotype, less root mass is available to forage for phosphorous at shallow depths (Lynch and Wojciechowski, 2015). Certain stresses tend to occur together often and therefore it might be useful to further investigate the specific RSA response to these conditions. A good example is salt stress and phosphate starvation, as phosphate ions tend to precipitate in saline soils and become unavailable to plants (Naidu and Rengasamy, 1993; Grattan and Grieve, 1998). Both stresses have contrasting effects on several RSA traits and the inhibiting effect of salt on lateral root development might even further limit phosphate uptake. Crucial in crop breeding aimed to optimize RSA is the availability of variation and plasticity in RSA, as observed among *Arabidopsis* accessions (Mouchel et al., 2004; Rosas et al., 2013; Ristova and Busch, 2014), related tomato species (Ron et al., 2013) and wheat varieties (Pound et al., 2013). Recently, Kawa et al. (2016) studied the natural variation in the response of 330 *Arabidopsis* accessions to the combination of salinity and phosphate starvation. In general, responses to salt stress were favored and especially lateral root growth was strongly inhibited. However, not all accessions showed the same response and this natural variation was associated with 13 genetic candidate loci for integrating the plants’ response to combined stress (Kawa et al., 2016). For many crops, however, the natural variation in RSA is currently still underexploited. Moreover, we need to advance our understanding of the adaptive value of genetically determined differences in RSA on the level of crop performance, marketable yield and fruit quality in targeted root environments and growth conditions.

Because the complexity of experiments and screenings increases with every additional variable, modeling can provide very useful tools to support research and breeding. A wide range of plant models on different scales is available to the community. These models should now be integrated with a multiscale modeling approach (Band et al., 2012; Rellán-Álvarez et al., 2016) in which developmental processes, RSA, outside environmental factors and plant performance are connected. Current models, however, are often not easy to integrate. When developing a model, the general challenge is to make it comprehensive, widely applicable and simple. For models describing RSA, most are falling short in one of these requirements. Some are only applicable for a certain species or stage of life, which limits the use for crop systems. As soon as models tend to be more widely applicable or incorporate more conditions, they tend to become more complex and the number of parameters increases. This decreases the ease of interpretation and especially the ease of integration into a larger model (including soil and plant performance models). The last few years, a range of more simple models have been published. These models are often based on a few simple rules. For example, ArchiSimple bases root system development on the fact that the growth rate of a root depends on the thickness of the root (Pagès and Picon-Cochard, 2014; Pagés et al., 2014). By using a simple and widely applicable model, it will be possible to implement models of soil behavior and of plant productivity. Some of the root models have already been integrated with models for changes in the soil (as reviewed in Pedersen et al., 2010; Dunbabin et al., 2013; Van der Putten et al., 2013) and show to be very promising in predicting the responses of the root system. One example is the ROOTMAP model, which integrates soil-water-nutrient dynamics with root growth responses in a three dimensional system (Dunbabin et al., 2002). Simulations are based on a simple external supply/internal demand principle. The model has shown its use in simulating the efficiency of different RSA types in both heterogeneous phosphate and nitrate supplies (Dunbabin et al., 2004; Chen et al., 2008). A good example of how such a model can provide valuable information is given by Chen et al. (2008), who show how the model can guide the efficient placement of phosphorus fertilizer. In a similar way, this kind of model could guide in selecting a preferred RSA and potentially even predicting possibly involved processes.

A model that integrates soil behavior, RSA and plant performance will offer a lot of information to breeders. To confirm whether a root system is advantageous under certain stresses as predicted by the model, RILS with contrasting root systems could be exploited (as illustrated in Liao et al., 2004; Zhu and Lynch, 2004; Zhu et al., 2005a). If indeed the predicted root system is advantageous, breeders could screen for this type of root system in a high throughput phenotyping system as described in the previous section. This screen can then be used for determining genes that are associated with this trait and can be used as targets for further selection. The model could also predict whether changing certain root system characteristics would negatively influence productivity. Of course, developing such a model is a major challenge still, but investments in developing a good model will be able to speed up crop selection and could model complex combinations of stresses.

### Challenge 3: Improving RSA without Compromising Yields

Crop selection on aboveground traits has lead to high-yielding cultivars and crop selection for a certain RSA may come with costs. The root:shoot ratio is known to increase during almost every abiotic stress that has been discussed in this review. On the other hand, selection on RSA does not equal selection for a bigger root system. Our examples show shifts between different root organs, rather than shifts in biomass partitioning between the shoot and the root. In this way, deeper rooting in rice, caused by expression of *HDG11*, confers drought tolerance without any yield penalty (Yu et al., 2013, 2016). However, unwanted side effects of selection are not uncommon. An excellent tool to address this problem is to make use of grafting.

Grafting is the process in which the root system (rootstock) of one plant is connected to the shoot (scion) of another (as reviewed in Warschefsky et al., 2016). This process naturally occurs in some tree species (Mudge et al., 2009) and this phenomenon may have triggered the development of grafting in Asia where it is now used in agriculture for over 2000 years to improve plant production (Kubota et al., 2008). In woody perennial crops (Albacete et al., 2015; Warschefsky et al., 2016) as well as in annual vegetable crops (Schwarz et al., 2010; Albacete et al., 2015), the selection and breeding of suitable rootstocks offers a powerful tool to sustain and expand the cultivation under suboptimal growth conditions (Gregory et al., 2013). Grafting has the advantage that not every cultivar needs RSA optimization separately, allowing improvement of rooting and (a)biotic stress tolerance of already existing elite cultivars. As such, grafting is considered as a surgical and fast alternative to breeding. Designing rootstocks for specific environments is becoming a feasible target to face future cultivation problems all around the world associated with global climate change (drought, salinization, occurrence of temperature extremes; Gregory et al., 2013). Important in this respect is to gain more knowledge of (i) the natural variation in RSA that exists within crops, and (ii) by what communication mechanisms the root(stock) modulates the shoot (scion) phenotype and performance, and visa versa (Warschefsky et al., 2016). In this way, grafting can rapidly advance our understanding of the adaptive value of differences in RSA on the level of shoot performance, marketable yield and fruit quality under targeted growth conditions.

## The Value of Model Species

A key aspect for engineering better performing crops via RSA optimization is improved understanding of the regulatory processes and underlying genetic components that regulate root growth. Root growth regulation, and its response to changing environmental conditions, is a highly complicated process that is controlled at many different levels by complex actions of gene networks in both time and space. Advances in this area are merely derived from work in *Arabidopsis* (as reviewed in Wachsman et al., 2015; Slovak et al., 2016). It is expected that due to the increasing number of highly efficient root phenotyping platforms, the use of GWAS for root traits, the increasing available functional genomics resources for roots, and the development of smart root model systems, much progress in our understanding of control mechanisms involved in root development will be achieved over the next 5–10 years.

Although *Arabidopsis* is often studied under artificial conditions, it is these conditions that make it possible to investigate the partly discussed mechanistic and cellular base behind the observed RSA responses. For crop species only limited information on these processes is available. Interestingly, most plasticity in RSA responses overlaps between our model species and crops, even independent of differences between monocots and dicots. Sparsely investigated functionality of RSA in *Arabidopsis* supports the results found in crops and conversely sparsely investigated molecular insights in crops confirmed results already established in *Arabidopsis*. Of course, not all mechanisms, responses and genes can be transferred from *Arabidopsis* to crops, but taken together the reviewed research, *Arabidopsis* proves to provide very valuable information for the development of crops able to withstand a wide range of abiotic stresses. This review stresses the importance of incorporating RSA into current crop selection, but we should not forget the wonderful tools we already have. Incorporating RSA into current crop selection also means incorporating *Arabidopsis* research into the current breeding pipeline, possibly even more then for aboveground traits.

## Author Contributions

All authors listed, have made substantial, direct and intellectual contribution to the work, and approved it for publication.

## Conflict of Interest Statement

The authors declare that the research was conducted in the absence of any commercial or financial relationships that could be construed as a potential conflict of interest.
